# Phytosanitary Interventions for Safe Global Germplasm Exchange and the Prevention of Transboundary Pest Spread: The Role of CGIAR Germplasm Health Units

**DOI:** 10.3390/plants10020328

**Published:** 2021-02-09

**Authors:** P. Lava Kumar, Maritza Cuervo, J. F. Kreuze, Giovanna Muller, Gururaj Kulkarni, Safaa G. Kumari, Sebastien Massart, Monica Mezzalama, Amos Alakonya, Alice Muchugi, Ignazio Graziosi, Marie-Noelle Ndjiondjop, Rajan Sharma, Alemayehu Teressa Negawo

**Affiliations:** 1 International Institute of Tropical Agriculture (IITA), Oyo Road, PMB 5320, Ibadan 200001, Nigeria; 2 The Alliance of Bioversity International and International Center for Tropical Agriculture (CIAT), Palmira 763537, Cali, Colombia; m.cuervo@cgiar.org; 3 International Potato Center (CIP), Avenida La Molina 1895, Lima 15023, Peru; j.kreuze@cgiar.org (J.F.K.); g.muller@cgiar.org (G.M.); 4 International Rice Research Institute (IRRI), Los Banos 4031, Philippines; g.kulkarni@irri.org; 5 International Center for Agricultural Research in the Dry Areas (ICARDA), Terbol Station, Zahle 1801, Lebanon; s.kumari@cgiar.org; 6 The Alliance of Bioversity International-CIAT & University of Liège, Gembloux Agro-BioTech, Passage des déportés, 2, 5030 Gembloux, Belgium; s.massart@cgiar.org; 7 International Maize and Wheat Improvement Center (CIMMYT), México-Veracruz, El Batán Km. 45, Texcoco 56237, Mexico; monica.mezzalama@unito.it (M.M.); A.Alakonya@cgiar.org (A.A.); 8 World Agroforestry Center (ICRAF), United Nations Avenue, Gigiri P.O. Box 30677, Nairobi 00100, Kenya; a.muchugi@cgiar.org (A.M.); i.graziosi@hotmail.it (I.G.); 9 Africa Rice Center (AfricaRice), 01 BP 2551, Bouake 99326, Côte d’Ivoire; M.Ndjiondjop@cgiar.org; 10 International Crops Research Institute for the Semi-Arid Tropics (ICRISAT), Patancheru 502324, Hyderabad, India; r.sharma@cgiar.org; 11 International Livestock Research Institute (ILRI), Addis Ababa P.O. Box 5689, Ethiopia; a.teressa@cgiar.org

**Keywords:** CGIAR, crop genetic resources, diagnostics, germplasm, crop breeding, pathogen, pest, Plant Treaty, phytosanitary regulations, transboundary pests, invasive species, prevention, quarantine, seed, seed health, virus indexing

## Abstract

The inherent ability of seeds (orthodox, intermediate, and recalcitrant seeds and vegetative propagules) to serve as carriers of pests and pathogens (hereafter referred to as pests) and the risk of transboundary spread along with the seed movement present a high-risk factor for international germplasm distribution activities. Quarantine and phytosanitary procedures have been established by many countries around the world to minimize seed-borne pest spread by screening export and import consignments of germplasm. The effectiveness of these time-consuming and cost-intensive procedures depends on the knowledge of pest distribution, availability of diagnostic tools for seed health testing, qualified operators, procedures for inspection, and seed phytosanitation. This review describes a unique multidisciplinary approach used by the CGIAR Germplasm Health Units (GHUs) in ensuring phytosanitary protection for the safe conservation and global movement of germplasm from the 11 CGIAR genebanks and breeding programs that acquire and distribute germplasm to and from all parts of the world for agricultural research and food security. We also present the challenges, lessons learned, and recommendations stemming from the experience of GHUs, which collaborate with the national quarantine systems to export and distribute about 100,000 germplasm samples annually to partners located in about 90 to 100 countries. Furthermore, we describe how GHUs adjust their procedures to stay in alignment with evolving phytosanitary regulations and pest risk scenarios. In conclusion, we state the benefits of globally coordinated phytosanitary networks for the prevention of the intercontinental spread of pests that are transmissible through plant propagation materials.

## 1. Introduction

### 1.1. International Germplasm Transfers for Food Security and Biodiversity Conservation

The international exchange of genetic resources, such as botanic seeds and vegetative propagules, has played a crucial role in agricultural and food diversification to an extent that about 68% of national food supplies are derived from crops with a foreign origin [1]. At the forefront of these international exchanges are the CGIAR genebanks, breeding and seed system programs that have made vital contributions for over five decades by assembling germplasm from all over the world for conservation, and adding value to those materials by characterizing, breeding, and making them available to users around the world [2,3]. (*Note: Germplasm used to denote plant propagation material, both true seed* (*orthodox, intermediate, and recalcitrant seeds*) *and vegetative propagules, from genebanks and breeding programs.*) Established in 1971, the CGIAR is part of the global agricultural research system, which makes critically important contributions to the United Nations Sustainable Development Goals (SDGs) in alleviating poverty and hunger and improving food and nutrition security and in the conservation of biodiversity [4].

The 11 CGIAR genebanks conserve over 760,467 accessions of cereals, grain legumes, forages, tree species, root and tuber crops, and bananas. These represent >174 genera and over 1000 species obtained from 207 countries, which are conserved in 35 collections around the world as seeds, in vitro material, and living plants in fields or screenhouses (Supplementary Table S1) [5]. Between 2007–2016, the CGIAR centers distributed 3.91 million samples, with about 30% from genebanks and 70% from crop breeding programs, to 163 countries [3,5]. These distributions from the CGIAR programs account for almost 89% of the total annual international germplasm exchanges *(note: ‘exchange’ and ‘transfers’ used as a common term to denote germplasm exports or imports between countries.*), under the International Treaty on Plant Genetic Resources for Food and Agriculture (ITPGRFA or the Plant Treaty) [2,3,6,7,8]. Between 2010 to 2019, the CGIAR genebanks acquired 116,921 distinct accessions, about 35% of which were acquired through the centers’ own breeding programs and 65% were acquired from collection missions or through national programs in 142 countries [7]. During the same period, the CGIAR genebanks distributed, on average, 115,000 samples of germplasm per year, and above 80% of the recipients were in developing countries [5,7]. A detailed analysis of the CGIAR genebanks’ acquisition and distribution of germplasm in the last decade is presented by Halewood et al. [7]. The demand for a global movement of plant genetic resources (PGR) from the international genebanks and breeding programs is increasing due to worldwide efforts to develop nutrient-rich high-yielding varieties, which are resilient to biotic and abiotic stresses and better adapted to a changing climate, through various programs, such as the CGIAR’s ‘Crops to End Hunger’ initiative [9]. Import and export of germplasm and other biological resources are influenced by several international and national policies, treaties, and legal frameworks [3]. The ITPGRFA and its multilateral system of access and benefit-sharing and the Convention on Biological Diversity (CBD) agreements guide the CGIAR centers’ policies on germplasm acquisition, conservation, regeneration, use, and distribution [3]. The availability of pest- and disease-free germplasm is an important requirement for international distribution from genebanks and breeding programs.

### 1.2. Pathogen and Pest Threats to International Germplasm Transfers

It is well-known that plants and seeds can harbor pathogens and pests (hereafter referred to as pest (*note: ‘pest’ used to denote any species, strain or biotype of plant, animal or pathogenic agent injurious to plants or plant products.*), including bacteria, fungi, phytoplasmas, viroids, viruses, insects, nematodes, and other harmful biotic agents, and that the transfer of germplasm carries a simultaneous risk of moving pests between geographies and introducing them into territories where they are not known to exist [10,11]. International seed transfers have been recognized as important pathways for the transboundary spread of pests through human activities associated with collection and distribution [10]. The threat may become severe, if more virulent strains or races of the pathogens are introduced [12]. Even pests with a low seed transmission rate, especially viruses, may lead to the development of an epiphytotic proportion of the disease in a field, if the other conditions (e.g., occurrence of insect vectors and susceptible hosts) and climate are favorable [13].

**Figure 1:**
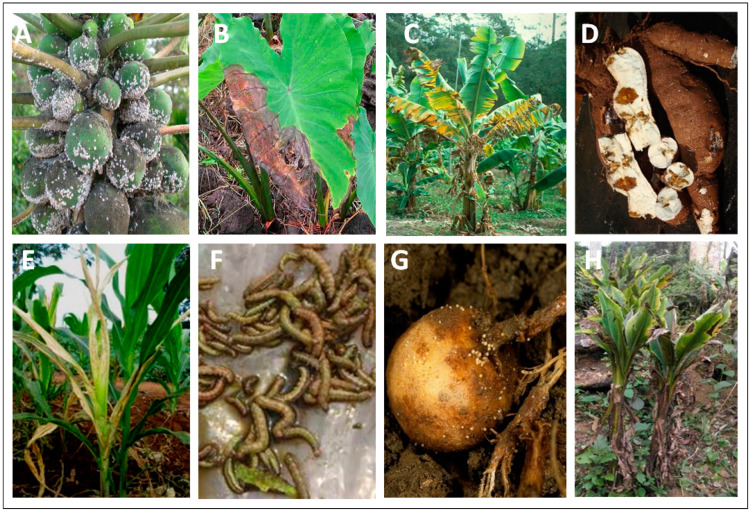
Examples of a few transboundary pests that have caused significant economic damage to food production and posed a major risk to germplasm transfers in sub-Saharan Arica. (**A**) Papaya mealybug (*Paracoccus marginatus*), (**B**) Taro blight (*Phytophthora colocasiae*), (**C**) Panama disease (*Fusarium oxysporum* Tropical Race 4), (**D**) Cassava brown streak (cassava brown streak ipomoviruses), (**E**) Maize lethal necrosis (Maize chlorotic mottle virus), (**F**) Fall armyworm (*Spodoptera frugiperda*), (**G**) Potato cyst nematode (*Globodera pallida*), and (**H**) Banana bunchy top (Banana bunchy top virus)

The introduction of economically important alien pests, a term used for non-indigenous pests introduced into new territory, from their centers of origin into new environments, has been reported in many different parts of the world [14]. Considering that every plant serves as a host for several insects and microbes of both a beneficial and harmful nature, every introduction of plant material is expected to result in the introducing exotic organisms. For instance, European farmers introduced wheat and its pathogens, *Mycosphaerella graminicola* and *Phaeosphaeria nodorum*, into the Americas, Australia, and South Africa in the past 500 years [13,14]. Some examples of introduced pests causing epidemics and pandemics with disastrous consequences for food production, livelihoods, and environmental biodiversity include the Irish potato famine in the 1840s caused by *Phytophthora infestans,* which was introduced from Central America into Ireland [11,15]. Some recent examples of devastating outbreaks caused by transboundary pest introductions into regions where the CGIAR operates include the maize lethal necrosis (MLN) epidemic in East Africa caused by maize chlorotic mottle virus (MCMV), which was introduced from East Asia [16]; the fall armyworm (*Spodoptera frugiperda*) outbreak in Africa and Asia, caused by the likely introduction of insect pest from the Americas [17]; the cassava mosaic disease outbreak in East Asia, caused by the Sri Lankan cassava mosaic virus (SLCMV), which was introduced from South Asia [18]; the banana bunchy top virus (BBTV) outbreak in the sub-region of Western Africa due the spread of the virus through planting material from the sub-region of Central Africa [19]; the expansion of banana wilt caused by the *Fusarium oxysporum* Tropical Race 4, which was introduced from Southeast Asia into South (India) and West Asia (Jordon and Israel), Mozambique and Colombia [20]; the outbreak of potato cyst nematode, *Globodera pallida*, in Kenya by the likely introduction of the pest from Europe [21]; the wheat blast outbreak in Bangladesh [22] and, more recently, in Zambia [23], caused by the *Magnaporthe oryzae* pathotype *Triticum,* introduced from South America; the spread of *Candidatus* Liberibacter solanacearum haplotype A and its vector, *Bactericera cockerelli* (potato psyllid), which is responsible for the potato Zebra chip disease and potato purple top disease, which are likely to have spread from the Central American region to Ecuador in South America [24]. The occurrence of these new pests in the territories was recognized during obvious disease outbreaks. A few examples of transboundary pests that have caused economically significant disease outbreaks through their spread to sub-Saharan Africa are indicated in Table 1 and Figure 1.

Many alien pests that could have caused significant damage were intercepted by quarantine authorities at the port of entry, preventing their introduction and establishment. For instance, in the USA, 7% of the 725,000 pest interception reports at the port of entry between 1984 to 2000 were attributed to plant propagative material [25]. In India, 45 viruses of quarantine importance were intercepted in imported plant germplasm between 1985–2016 [26]. The number of first reports of crop pests in new hosts and/or new regions has increased in recent years, driven by agricultural intensification, international trade, and climate change [13,27]. An analysis of 1300 known invasive pests and pathogens estimated their potential cost to global agriculture at over US$ 540 billion per year, if they continue to spread [28,29]. National capacities to prevent and manage alien pests are sub-optimal in much of Africa, as well as in parts of the Latin America, the Middle East, Central Asia, and Indochina, predisposing global biodiversity hotspots in these regions to the risk of exotic pest invasion [30]. Increasing risk of pest introduction and limited capacity to act against invasions in most countries warrant robust strategies to prevent transboundary pest spread, especially through propagation material, as such strategies decrease the chance of pest introduction, establishment, and further spread.

### 1.3. Transboundary Pest Risk to Germplasm Distribution and Premises for the Establishment of CGIAR Germplasm Health Programs

The nature of the CGIAR germplasm acquisition and distribution operations presents a high-risk scenario for transboundary pest spread, consequently, the safety of germplasm movement has been a major concern. The main reasons for this are the diverse origin of accessions, which are acquired from different geographies that converge at a research station for regeneration and characterization, and their distribution to diverse locations [7,15]. For instance, in the past 10 years, CGIAR genebanks have distributed 854,000 samples to 150 countries, at an average of 105,000 samples per year, catering for about 2000 requests from 100 countries [5]. The risk of exotic pest introduction through an accession into a new territory and its spread in the case of favorable environmental conditions is high. Similarly, endemic pests in regeneration sites can migrate to new territories along with the germplasm. Poor phytosanitary management also has detrimental effects on the survival of accessions during regeneration and evaluation, and it lowers the viability of the germplasm in the conservation facility, and it could lead to a loss of diversity in the collections and genetic erosion [31].

Without the proper phytosanitary measures of international agencies, such as the CGIAR, germplasm distribution increases the possibility of pest dissemination in areas previously considered to be disease-free [32,33]. Unintended pest spread through germplasm is a significant concern for the CGIAR genebanks and breeding programs, the majority of which distribute germplasm to developing countries and biodiversity hotspots that lack a sufficient quarantine capacity to prevent pest entry or respond to pest outbreaks [30]. Recognizing these pest risks, the CGIAR centers have setup Germplasm Health Units (GHUs), with the objectives of (i) averting the spread of quarantine pests with CGIAR germplasm transfers, (ii) preventing pest outbreaks, (iii) safeguarding biodiversity, and (iv) strengthening the development of phytosanitary capacities. The GHUs ensure compliance with the Food and Agriculture Organization (FAO)-International Plant Protection Convention (IPPC) procedures, which have the force of a legal treaty, and are enforced by the National Plant Protection Organization (NPPO or quarantine agency) to regulate pest spread through transfers of germplasm [34]. The movement of germplasm internationally is subjected to the same rules, with the directive that germplasm should be free of regulated pests for safe transfers across international boundaries [35]. Therefore, it is crucial to test the health of germplasm accessions before distribution and before it is used planting material. As the Center’s liaison, GHUs engage with the NPPO of the host and recipient countries to organize import permits, conduct inspections of regeneration fields, conduct germplasm health testing and phytosanitation, and prepare germplasm for exportation or importation in accordance with the International Standards for Phytosanitary Measures (ISPMs) of the IPPC and other recommended actions, including the FAO-International Board for Plant Genetic Resources (IBPGR) technical guidelines for the safe movement of germplasm [36].

In this paper, we describe the mission and functions of the CGIAR GHUs and how transdisciplinary approaches are adopted for the phytosanitary protection of germplasm at all stages of the value chain, from acquisition for conservation in genebanks to regeneration for accession increase, breeding, safety duplication and regional and international distribution for the safe global movement of germplasm from the 11 CGIAR genebanks and breeding programs. We present the emerging phytosanitary challenges and the increased risk of transboundary pests and pathogens to the international exchange of germplasm, followed by lessons learned and recommendations stemming from experience of the international network of GHUs, which operate across all continents in collaboration with the NPPOs and plant health organizations. We conclude by stating the benefits of globally coordinated phytosanitary networks to prevent the transboundary spread of diseases through plant propagation materials.

## 2. Historical Evolution of GHU and Its Core Functions

### 2.1. Development of Institutional Capacity for the Prevention of Transboundary Pest Spread through Germplasm

The first set of plant quarantine procedures, as a legal measure, was established in 1873 in Germany to regulate potato tuber imports from the USA in order to prevent the spread of the Colorado potato beetle, *Leptinotarsa decemlineata*. The first international agreement on measures to control the pests through regulation of the movement of plants was established in 1878 as an “International Convention on Measures to be taken against *Phylloxera vastatrix* (present name *Viteus vitifoliae*)”, an insect pest that was introduced with the vine cuttings imported from the USA to France in 1865 [14]. This agreement between seven European countries, which came into force on 3 November 1881, has specified procedures for the certification of plant material for export and import in order to control grapevine phylloxera [14]. Many countries subsequently followed suit by imposing quarantine regulations to contain the spread of pests through plants and plant products, which led to the establishment of the International Convention for the Protection of Plants in 1929 by the International Institution for Agriculture in Rome. The International Plant Protection Convention (IPPC), adopted by the sixth Conference of the FAO in 1951, which came into force on 3 April 1952, was eventually established as a multilateral treaty on plant protection and replaced previous agreements [34]. The IPPC and the 1994 World Trade Organization (WTO) agreement on the Application of Sanitary and Phytosanitary (SPS) Measures (the SPS Agreement) shaped international plant quarantine policy and standards for regulatory measures implemented by member countries for the protection of plants, animals, and human life [34]. However, the implementation of quarantine procedures differed all over the world, with many developing and underdeveloped countries unable to implement adequate measures, due to poor resources and a lack adequate physical and technical capacity [28,37].

At the time of the establishment of the early CGIAR centers—the IRRI, CIMMYT, CIAT, and IITA—in the 1960s, the subsequent formation of the CGIAR and the establishment of new centers, such as CIP, ICARDA, and ICRISAT, in 1971, many NPPO institutions were under development. In the early stage of the CGIAR, international germplasm exchange programs faced multiple challenges due to a lack of adequate baseline knowledge on pests affecting its mandate crops and weak quarantine infrastructure in many countries in which they were operating [38]. For instance, IITA depended on an intermediary quarantine station in the Netherlands for the importation of cassava from outside Africa in the 1970s and 1980s [15]. Similarly, the INIBAP (International Network for the Improvement of Banana and Plantain) Transit Centre (ITC) was established in 1985 by INIBAP at the Universiteit Leuven in Belgium as a transit center for *Musa* collection. The “INIBAP” was replaced by “International” when INIBAP and IPGRI (International Plant Genetic Resources Institute) were merged to establish Bioversity International at the end of 2006 [39].

To address the pest risks associated with the CGIAR germplasm exchange activities, a task force was established in 1975 by the IBPGR, which led to the publication of “Plant Health and Quarantine in the International Transfer of Genetic Resources” [40], outlining the control actions required to address the seed health challenges encountered by International Agricultural Research Centers (IARCs). This was followed by a series of consultations in the following decade, and an informal recommendation by the Regional Plant Protection Organizations (RPPOs) in 1988, which fostered an FAO and IBPGR joint program to facilitate the safe exchange of germplasm, including the drafting of technical guidelines for the safe movement of germplasm for major crops [36]. A CGIAR commissioned study in 1989 on “Plant Quarantine and the International Transfer of Germplasm” recognized a lack of accurate, up-to-date information on pests and poor accessibility to updated information by national quarantine officials to be among the main problems, and the study recommended that CGIAR centers adopt standardized phytosanitary procedures for germplasm transfers [37]. This led to the foundation of an “inter-center collaboration on germplasm health and exchange”, and the first meeting of the pathologists and virologists in charge of germplasm health from the CIMMYT, CIP, IBPGR, ICARDA, ICRISAT, IITA, and IRRI, which was convened by the IBPGR in Rome on October 1990 as a formal meeting of the germplasm health program [41]. In 1993, following the recommendations of the Sixth International Plant Protection Congress held in Montreal in August 1993, the CIMMYT, CIP, ICARDA, ICRISAT, IITA, and IRRI established GHUs as independent units within the centers to undertake research and facilitate the safe exchange of PGR from genebanks and breeding programs. Similar programs were subsequently established in the remaining five centers. These programs, differently named the Germplasm Health Unit (AfricaRice, Bioversity, CIAT, ICRAF, IITA, and ILRI), Seed Health Unit (CIMMYT, ICARDA, and IRRI), Health Quarantine Unit (CIP), and Plant Quarantine Unit (ICRISAT), serve the same mission of preventing phytosanitary risks associated with CGIAR germplasm activities and ensuring the safe transfer of germplasm.

### 2.2. GHUs as CGIAR Gateway for Safe Germplasm Exchange

The GHU mission is to maintain the multidisciplinary capacities required for health testing, ensuring the implementation of phytosanitary procedures to eliminate pests, facilitate the production of pest-free germplasm, and make “go/no-go” decisions on germplasm transfers from the centers based on phytosanitary statuses. The six strategic objectives of GHUs are: (i) to ensure that the transboundary movement of germplasm and non-seed biological materials complies with the regulatory guidelines of the importing and exporting countries and that the materials are free of quarantine pests; (ii) to develop and adopt phytosanitary procedures to generate pest-free germplasm; (iii) to develop diagnostic tools for seed health monitoring and pest surveillance; (iv) to conduct pest risk assessments of germplasm activities, including conservation, seed increase, and transfers; (v) to contribute to the development of phytosanitary capacity; and (vi) to organize a GHU Community of Practice to form a network of centers in transboundary pest prevention.

From the moment of their establishment, these centers began to develop expertise on the safe exchange of germplasm for its mandate crops by mobilizing interdisciplinary capacities for seed and plant health testing, phytosanitation, and therapy procedures in order to generate pest-free planting material or salvage germplasm after eliminating the risk of contaminating pests [36,41]. This evolution was extremely challenging due to the inadequate knowledge on pests affecting crop species of interest in operational territories. This has often led to the task of simultaneously conducting research on the identification and characterization of pests, developing diagnostics for pest detection, and establishing procedures for germplasm phytosanitation [15]. In this process, the CGIAR centers have worked closely with the NPPOs of the host countries to standardize safe germplasm exchange procedures, which were eventually transformed into a formal collaboration between the center and the host country NPPOs, leading to special accords for CGIAR germplasm transfers. For instance, in 1978, the Indian Council of Agricultural Research (ICAR) accorded permission to set up an “Export Certification Quarantine Laboratory” (Plant Quarantine Unit) at the ICRISAT headquarters in Patancheru, Hyderabad, India [38]. The need for adaptive changes remains a consistent requirement for coping with the changing phytosanitary risks in the world. For instance, the MLN outbreak in East Africa led to the redrafting of safe maize exchange procedures [42], and the characterization of casual viruses in the 2000s of cassava brown streak disease, described in the 1920s, led to the drafting of a new protocol for cassava virus indexing and the production of virus-free cassava planting material [43], which was followed by a reindexing of the cassava collection held in the IITA Genetic Resources Center at Ibadan, Nigeria. GHU programs have demonstrated a high commitment to the minimization of the pest risks associated with germplasm transfers as many national programs, especially in sub-Saharan Africa, lack sufficient facilities to carry out the required testing and/or phytosanitation of the accessions before their release for propagation use [30]. GHUs perform all their activities in close collaboration with NPPOs, RPPOs, and several national and international plant health programs. The centers’ policy mandate for all germplasm, outgoing and incoming, is channeled and cleared through GHUs to ensure safe import or export, and GHUs have, over the years, developed into the centers’ gateway for the safe international exchange of germplasm.

In 2017, GHUs were aligned with the Germplasm Health (GH) component of the CGIAR Genebank Platform [5]. This offered a unique opportunity for strengthening collaboration among GHUs, catalyzing a harmonized CGIAR GHU strategy, adopting a common Quality Management System (QMS) to ensure uniform standards across the centers, and the implementing cross-center R4D initiatives to address recalcitrant and emerging phytosanitary challenges. These recent developments, in close collaboration with NPPO partners, resulted in GHUs becoming a global network for transboundary pest prevention and effectively addressing the emerging needs of CGIAR programs.

## 3. Procedures for Germplasm Health Testing and Safe International Transfers

### 3.1. Multistage Phytosanitary Controls for Pest Prevention

The multidisciplinary and multistage process of GHUs for ensuring the phytosanitary safety of bioresources has five stages [15]: (i) germplasm health testing for pests using a range of diagnostic methods, including conventional bioassays, culturing methods, serological detection using enzyme-linked immunosorbent assay (ELISA), and nucleic acid-based detection (nucleic acid hybridization techniques, or various formats of DNA and RNA amplification, including polymerase chain reaction (PCR) and isothermal amplification methods, such as loop-mediated isothermal amplification (LAMP), and recombinase polymerase amplification (RPA), or sequence-independent high-throughput sequencing (HTS) and bioinformatics virus detection [44]); (ii) physical inspection to eliminate infected and physically damaged true seeds and vegetative propagules [12]; (iii) pest risk mitigation during germplasm regeneration using the most optimum procedures, including inspection of plants during the active growth stage, the use of pesticides and weed management in the field, nursery, and screenhouse production sites, and the use of virus-free planting material for clonally propagated germplasm [36]; (iv) phytosanitation (treatment) of germplasm, as a curative procedure to eliminate pests and salvage germplasm [36]; and (v) documentation for traceability and regulatory compliance, which includes an import permit issued by the NPPO of the import country that enlists phytosanitary conditions for import qualification; a phytosanitary certificate issued by the NPPO of the export country, ensuring that the germplasm complies to conditions listed in the import permit; and a health statement issued by GHUs, with a description of the germplasm origin, propagation, and health assessment information [36].

**Figure 2:**
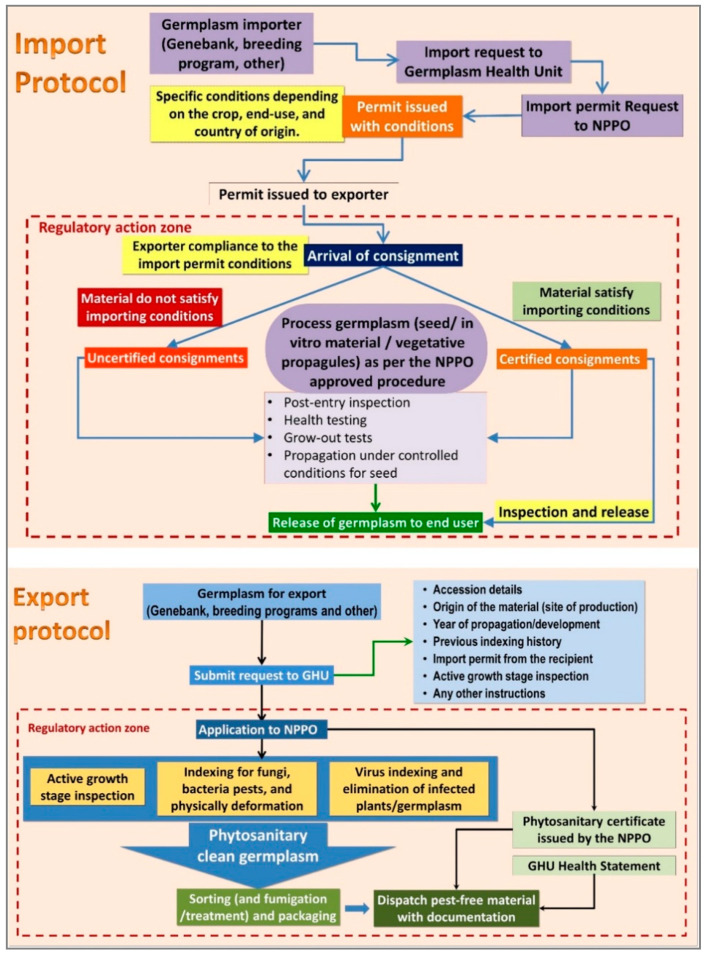
Schema of the germplasm import and export protocol of the CGIAR centers, which is executed in collaboration with the host country national plant protection organization (NPPO). The activities conducted under NPPO monitoring are marked as the ‘regulatory action zone’. Permit = an official document issued by the NPPO authorizing the centers to transfer materials; country of origin = country in which the germplasm is regenerated.

Generally, CGIAR centers acquire (imports) or distribute (exports) small quantities of germplasm as a few grams of botanic seeds or a small number of vegetative propagules of accession as in vitro plantlets, tubers, corms, or stem cuttings. All countries regulate incoming genetic resources according to the national and international laws and regulations, which are designed to prevent the risk of pest introduction. It is necessary for the CGIAR centers to align their phytosanitary compliance procedures with the host country NPPO, which is the statutory organization that sets policies, laws, and regulations to oversee plant material transfers according to the IPPC framework and agreements. The import and export of germplasm is a collaborative endeavor between the GHU and the NPPO of the country of export and import, the germplasm provider, and the germplasm material recipient (Figure 2). The GHUs submit imported germplasm to the NPPO of the host country for post-entry inspection to ensure compliance with the importation conditions, including checks for quarantine pests in the post-entry testing facility or regeneration of germplasm in a quarantine isolation area, prior to the release of safe material to the requester. A similar procedure is used to export germplasm from the centers. The GHUs conduct specific checks, as indicated on the import permit, and dispatch the material with or without seed treatment, depending on the importer’s requirements.

### 3.2. Criteria for Pest Monitoring

A wide variety of pests is reported for each crop species, some of which are ubiquitous with the host species distribution in all geographies, and some pests are restricted to a few geographies [45]. Monitoring germplasm for pests depends on the knowledge of the pests affecting a crop species, particularly in the country regenerating the accessions, the crop propagation method (true seed or vegetatively propagation), and the ability of a pest to spread through germplasm. On the basis of the pest biology of different crop species and the economic significance and risk associated with crops and production systems, the NPPOs of each country have established national pest lists that categorize pests as “unregulated” or “regulated pests”, with a further division of regulated pests into “quarantine pests” and “regulated non-quarantine pests” [46]. Pests whose introduction into an area can result in severe destruction are classified as quarantine pests. Regions can be free of quarantine pests, or such pests may exist but not be widely distributed (e.g., cassava brown streak virus, *Fusarium oxysporum* f. sp. *cubense* Race 4, and *Candidatus* Liberibacter solanacearum). Quarantine pests are strictly controlled through official monitoring measures, which are enforced by the NPPO. However, regulated non-quarantine pests are widely distributed, and their presence in germplasm causes planting material losses or initiates new disease cycles (e.g., cucumber mosaic virus). Unregulated pests include endophytes, saprophytes, and other pests of no significance.

The pest categorization and country-specific lists of quarantine and regulated non-quarantine pests are established by the NPPO. They are dynamic lists, which are updated regularly [47]. Countries and regions also use alternative classifications to designate regulated and unregulated pests. For instance, the European Plant Protection Organization (EPPO) uses “A1 pests” and “A2 pests” based on the complete absence or presence of designated pests in the EPPO region, respectively, and the Nigerian Agricultural Quarantine Services (NAQS) classifies pests under three categories: Category A (quarantine pest) for pathogens that are not present in Nigeria and/or in any country in West Africa; Category B (restricted regionally occurring pest) for pathogens that have a restricted local distribution in Nigeria and/or West Africa, against which field inspection and/or seed health testing methods can provide adequate protection; and Category C (regulated non-quarantine pests) for internationally widespread pathogens that may affect seed quality [15].

National plant pest lists provide information on pests likely to be associated with a plant species in the country of origin, and national regulated pest lists provide information on pests that need to be controlled using rigorous quarantine measures. Both these types of pest lists form the basis for setting the conditions of germplasm transfers between countries. The IPPC has established standards and frameworks for preparing a regulated pest list [48], and pest risk assessment (PRA) procedures for establishing scientifically justified regulations for the prevention of regulated pest incursions [48,49]. However, such analyses are often limited to commercially traded crops (e.g., chickpea, groundnut, maize, potato, rice, sorghum, soybean, and wheat), and the information on the pest occurrence, economic significance, distribution, and epidemiology is scanty or non-existent for several minor and orphan crops, as well as wild relatives. In addition, changes to pest nomenclature, due to taxonomic revision, which necessitates additional efforts to revise the pest lists, further complicates compliance procedures. Many countries do not update pest lists regularly, and the fact that importation or exportation conditions are based on outdated pest lists poses a challenge for regulatory compliance. Considering the variations and gaps in the country-specific lists due to limited knowledge of the pests affecting crop species, GHUs have taken a standardized approach to conducting checks for all quarantine and regulated non-quarantine pests reported for each species (detailed in Section 4). These procedures for the minimization of the risk of the spread of known and unknown pests through germplasm are in line with the FAO-IBPGR Technical Guidelines for the Safe Exchange of Germplasm [36], ISPMs, and other best practices [50].

## 4. Germplasm Health Testing and Pest Elimination

Seed health testing and pest detection is a first-line approach in managing seed-borne and seed-transmitted pests. In the case, of true seed crops, some pests infecting host crops are seed-borne (e.g., *Fusarium oxysporum* in cowpea), some are seed-transmitted (e.g., bean common mosaic virus in cowpea and common bean), and some are either seed-borne or seed-transmitted (e.g., *Phyllachora maydis,* which is responsible for tar spot affecting maize) [10,51]. Seed-borne and seed-transmitted pests are a concern for germplasm conservation and exchange, and procedures are therefore used to eliminate pests including the use of seed treatment methods or the regeneration and harvesting of seed from healthy plants. However, most pests affecting vegetatively propagated crops, especially intracellular pests, such as phytoplasmas, viruses, and viroids, can spread through vegetative propagules, and eliminating them requires the use of complex procedures. The GHUs routinely check for about 320 pests that are endemic in germplasm production sites, including bacteria, fungi, insects, nematodes, oomycetes, phytoplasmas, viruses, and viroids (Supplementary Table S2). The testing also covers other pests listed in the import permit of the country that receives germplasm. According to the crop mandate of the center, each GHU is specialized in enabling the production of quality germplasm in accordance with the best procedures available for the diagnosis and detection of pests, treatment for phytosanitation, and international transfers. GHUs apply similar procedures for genebanks and breeding programs, although genebank materials are more diverse, including wild species, landraces, and new acquisitions from new collection missions, which may demand complex/time-consuming procedures, owing to the different species biology and pest risks. The breeding program materials mostly comprise staple cereals, grain and oil seed legumes, roots, tubers, and banana crops. In general, managing the phytosanitary risks associated with true seed crops is relatively easy and effective, as not all the pathogens and pests are seed-transmitted, or seed-borne. Moreover, it is relatively easy to control or eliminate infections of seed-transmitted, and seed-borne pests from seed using chemical or heat treatments, thus salvaging germplasm. In the case of clonally propagated crops, however, systemically infectious pathogens, especially viruses and viroids, are difficult to eliminate without applying complex procedures, which are expensive and time-consuming. Brief details on the procedures employed to generate pest-free germplasm by crop group are summarized here.

### 4.1. True Seed Crops

#### 4.1.1. Cereals

Cereal germplasm from breeding programs and genebanks is inspected for both seed-borne and seed-transmitted pests. General procedures for testing, detection, diagnosis, and seed treatment for the elimination of seed-borne pests are used [51,52], including the International Seed Trade Association (ISTA) methods, where applicable [53]. The general phytosanitary procedures used for true seed phytosanitation include, (i) active-growth stage inspection at the flowering/pre-harvest stage to check for the presence of any regulated pests and seed-transmitted pests; (ii) dry seed examination using a desk magnifier (2x) to remove the admixtures of plant debris, sclerotia, galls, insects, smut sori, and discolored and moldy seeds; (iii) seed-washing and a sedimentation test to detect the spores that could not be detected either in dry seed examination or incubation tests; (iv) standard blotter tests to detect the presence of fungi; (v) an agar test (selective media) to detect the bacterial pathogens using specific media; (vi) a seed soaking test to detect the presence of nematodes; (vii) a seed treatment involving a fungicidal treatment to remove saprophytic fungi and seed-borne pathogens; and (viii) seed fumigation using aluminum phosphide (or methyl bromide for sorghum seeds, as per the requirement of Indian NPPO at ICRISAT, India) [54]. The tests performed for some important seed-borne and seed-transmitted diseases of various CGIAR mandate crops are summarized below.

*Barley:* The most important seed-borne fungi are smut (*Ustilago nuda*), covered smut (*Ustilago hordei*), spot blotch (*Bipolaris sorokiniana*), head blight (*Fusarium graminearum*), barley leaf stripe (*Pyrenophora tritici-repentis*), ergot (*Claviceps purpurea*), a bacterium responsible for basal glum rot (*Pseudomonas syringae* pv. *atrofaciens*); a virus (barley stripe mosaic virus (BSMV)), a seed gall nematode (*Anguina tritici*), and an insect, the khapra beetle (*Trogoderma granarium*). Standard phytosanitary procedures are used to test and generate pest-free germplasm for import and export, including considerations of the additional conditions laid down by the NPPO of the import and export countries.

*Maize:* The main risks associated with maize germplasm exportation are associated with pathogens, such as *Pantoea stewartii* pv. *stewartii* maize dwarf mosaic virus, maize chlorotic mottle virus, sugarcane mosaic virus, and wheat streak mosaic virus, which have a restricted geographical distribution. These pathogens are proven to be seed-borne and seed-transmitted, although some of them have a low transmission rate of <1%. Many other maize pathogens are listed in the requirements of the country importing the germplasm, and the measures taken to guarantee that seeds are pathogen-free cover a wide range of possible threats by applying strict phytosanitary procedures in the multiplication field plots and exhaustive laboratory seed testing using conventional, serological, and molecular methods and seed treatments.

*Rice:* Many pests and pathogens have been identified as posing a risk to rice germplasm. GHUs use various procedures, as summarized above, for seed-borne pests, including bacteria (*Psudomonas* spp., *Xanthomonas* spp.), fungi (*Magnaporthe oryzae, Tilletia barclyayana*, etc.), oomycetes (*Sclerophthora macrospora*), phytoplasma (*Candidatus* phytoplasma 16srIII-L), virus (rice yellow mottle virus) and nematode (*Aphelenchoides besseyi*) on seeds.

*Sorghum and millets:* Some of the important sorghum seed-borne diseases are ergot (*Claviceps sorghi*), anthracnose (*Colletotrichum graminicola*), leaf blight (*Exserohilum turcicum*), downy mildew (*Peronosclerospora sorghi*), loose kernel smut (*Sporisorium cruentum*), long smut (*S*. *ehrenbergii*), head smut (*S*. *reilianum*), covered kernel smut (*S*. *sorghi*), bacterial blight (*Ralstonia andropogoni*), bacterial leaf streak (*Xanthomonas vesicola* pv. *holcicola*), and bacterial leaf spot (*Pseudomonas syringae* pv. *syringae*). Ergot (*Claviceps fusiformis*), and smut (*Moesziomyces penicillariae*) are the major seed-borne diseases of pearl millet. There are also some reports of downy mildew (*Sclerospora graminicola*) being seed-borne in nature. *Melanopsichium eleusinis, Pyricularia grisea*, and *Bipolaris* sp., are the important pathogens of small millet, for which salvaging treatment is used to recover pest-free seeds.

*Wheat:* The main risks associated with the germplasm exportation of bread and durum wheat are associated with pathogens, such as Karnal bunt (*Tilletia indica*), common bunt (*T. tritici* and *T. laevis*), *Alternaria triticina*, *Xanthomonas translucens* pv. *undulosa*, BSMV, and wheat streak mosaic virus (WSMV), which have a restricted geographical distribution. Nevertheless, many more wheat pathogens are listed in the requirements of the country importing the germplasm, and the measures taken to guarantee that the seeds are pathogen-free cover a wide range of possible threats by applying strict phytosanitary procedures in the multiplication field plots and exhaustive laboratory seed testing and seed treatments. Germplasm that is imported is subject to the NPPO regulations and inspected very carefully for wheat blast (*Magnaporthe oryzae* pathotype *Triticum*), dwarf bunt (*T. controversa*), and flag smut in wheat (*Urocystis agropyri*). In addition to the fungal pathogens, inspections are also carried for seed-borne insect pests, such as *T. granarium* (the khapra beetle), in seed exports and imports.

#### 4.1.2. Grain and Oil Seed Legumes

Legume germplasm is more prone to pest attack, and many of these pests are known to spread through seeds [45]. A list of regulatory pests and pathogens frequently tested in the legume germplasm regeneration sites of CGIAR is given in Supplementary Table S2. The stringent phytosanitary and seed health testing procedures, such as those described for cereals, are also applied for legumes to prevent the transfer of fungal, bacterial, and viral diseases through legume germplasm. In general, germplasm and breeding lines for international transfers are regenerated under screenhouse conditions to avoid viral infections, and the germinated plants are inspected for viral symptoms and indexed by ELISA or PCR-based methods to ensure that plants are free from viruses prior to seed harvesting. Grow-out tests under screenhouse conditions are performed to assess seed-transmitted viruses, which is a standard practice for legumes. Although it is a time-consuming procedure, but it offers a reliable detection that eliminates the risk of viruses. Some of the important seed-borne and seed-transmitted pests, for which observations are conducted for export and import quarantine, are listed by crop below.

*Bean:* About 23 seed-borne bacterial, fungal, and viral pathogens are reported to be important for beans, including common blight (*Xanthomonas campestris* pv. *phaseoli*), charcoal rot (*Macrophomina phaseolina*), and anthracnose (*Colletotrichum truncatum*), along with three seed-transmitted viruses (alfaalfa mosaic virus (AMV), bean common mosaic virus (BCMV), and peanut mottle virus (PeMoV)).

*Cowpea, bambara groundnut and other* Vigna *species*: Several fungi and bacterial pathogens of cowpea are seed-borne, including cowpea bacterial blight (*Xanthomonas axonopodis* pv. *vignicola*), web blight (*Rhizoctonia solani*), and brown blotch (*Colletotrichum capsici*). About 10 viruses are reported to be seed-transmitted in cowpea. The most frequent viruses of interest in seed transmission are cucumber mosaic virus (CMV), cowpea yellow mosaic virus (CYMV), cowpea mottle virus (CmeV), southern bean mosaic virus (SBMV), and cowpea mild mottle virus (CPMMV). Cowpea seeds are subjected to fumigation with phostoxin (55% aluminum phosphide) to eliminate insect pests and treated with fungicide to eliminate seed-borne pathogens.

*Chickpea and pigeonpea*: Important seed-borne diseases of these two grain legumes are blight (*Ascochyta rabiei*), grey mold (*Botrytis cinerea*), wilt (*Fusarium oxysporum* f. sp. *ciceri*), and stem blight (*Phomopsis longicolla*) in chickpea; blight (*Botryodiplodia theobromae*) and wilt (*Fusarium oxysporum* f. sp. *udum*) in pigeonpea.

*Faba bean*: Twenty fungal species belonging to 13 genera were recognized as seed-borne risk (*Aspergillus*, *Penicillium*, *Alternaria*, *Botrytis*, *Cephalosporium*, *Cladosporium*, *Epicoccum*, *Fusarium*, *Rhizoctonia*, *Rhizopus*, *Stemphylium*, *Trichothecium*, and *Verticillium*), along with four seed-transmitted viruses [broad bean stain virus (BBSV), bean yellow mosaic virus (BYMV), broad bean mottle virus (BBMV), and pea seed-borne mosaic virus (PSbMV)]. Broomrape (*Orobanche* and *Phelipanche* spp.), root parasitic weeds, are also considered to pose a threat and measures are taken to avoid germplasm multiplication in the broomrape infested fields.

*Groundnut*: Dry root rot (M. phaseolina/Rhizoctonia bataticola), root rot (Rhizoctonia solani), pod rot (Sclerotium rolfsii), Sphaceloma arachidis (groundnut scab), *Ralstonia solanacearum* (African strains), seed bruchid (Stator pruininus), Testa nematode (*Aphelenchoides arachidis*), peanut mottle virus (PMV), peanut stripe virus (PStV), peanut clump virus (PCV), Indian peanut clump virus (IPCV), peanut stunt virus (PSV), and tobacco streak virus (TSV) are the important quarantine pests for groundnut.

*Lentil***:** The important fungal seed-borne diseases of lentil include *Ascochyta lentis* (ascochyta blight), and *Fusarium oxysporum* f. sp. *lentis* (fusarium wilt), botrytis grey mold (*Botrytis fabae* and *B. cinerea*), Stemphylium blight (*Stemphylium botryosum*), phoma blight (*Phoma medicaginis* var. *medicaginis*), and anthracnose (*C. lindemuthianu* and *C. truncatum*); stem nematode (*Ditylenchus dipsaci*), and seed-transmitted viruses, include, AMV BYMV, PSbMV, CMV, and BBSV.

*Soybean*: The seed-borne fungal and bacterial pathogens of soybean are soybean bacterial pustule (*X. axonopodis* pv. *glycinea*), brown spots (*Septoria glycinea*), frogeye leaf spots (*Cercospora sojina*), yellow leaf spots (*P. manshurica*), charcoal rot (*M. phaseolina*), and anthracnose (*C. truncatum*). Many seed-transmitted viruses are also reported in soybean, including BCMV, CMV, CYMV, CmeV, CPMMV, and SBMVin West Africa. Rigorous tests are also conducted for other viruses depending on the country of origin. Seeds are fumigated with phostoxin (55% aluminum phosphide) to eliminate insect pests and fungicide treatments are given to eliminate seed-borne pathogens.

### 4.2. Vegetatively Propagated Crops

Banana (and plantain), cassava, potato, sweetpotato, and yam are the major vegetatively propagated crops (VPCs) exchanged by the CGIAR programs [55]. Vegetatively propagation poses the greatest risk of the introduction of pests through planting material, which can carry any infections from previous seasons to the next cropping cycle and thus accumulate pathogens, especially viruses, over generations of cultivation. Many transboundary pest introductions have been linked with the transfer of vegetative propagules: the spread of BBTV to Africa and its further spread in the continent [19,56]; in the case of potato, the necrotic strains of potato virus Y (PVY) in Brazil and aggressive strains of potato late blight in Africa and Asia and potato cyst nematode (*Globodera palladi*) in East Africa; in the case of cassava, the regional spread of cassava brown streak virus (CBSV), which is attributed to contaminated stem propagation; and in Asia, the spread of Sri Lankan cassava mosaic virus (SLCMV) from South Asia to East Asia [57]. Therefore, many countries regulate vegetative germplasm importation, and the FAO-IPGRI technical guidelines recommend that only in vitro plants that have been tested for pathogens should be moved between countries [36]. The pollen or true seed of these crops are also exchanged for breeding purposes under adequate phytosanitary controls. By limiting international movement to sterile in vitro plants, the only concern that remains is intracellular obligate pathogens, such as viruses, viroids, and phytoplasmas. Depending on the country, some viruses are regulated by quarantine procedures (e.g., BBTV, CBSV, and PVY), and several other viruses are unregulated (e.g., sweet potato mild mosaic virus). Nonetheless, the standard procedure used by GHUs includes the generation of virus-free in vitro plants as per the FAO/IBPGR technical guidelines for the conservation and distribution of these crops [36]. All the material exported and imported are tested for viruses, and other pests under NPPO guidance, and only material free of viruses, and other pests, is released to the end-users. Unlike cereals and legumes, the phytosanitation, and testing procedures for clonally propagated crops differ according to the crop species, as explained below.

*Banana:* Banana is a perennial herbaceous plant, traditionally propagated using suckers (side shoots generated from underground corms), and is thus often carries both soil-borne insects and fungi, in addition to shoot-invading viruses, fungi, and bacterial agents. Several banana pathogens, like *Fusarium oxysporum* f. sp. *cubense* tropical race 4 (Panama disease), banana Xanthomonas wilt (*Xanthomonas campestris pv. musacearum*), and several viruses, such as BBTV and banana bract mosaic virus (BBrMV) have restricted geographic distribution. Guaranteeing the movement of pathogen-free germplasm is an important task to minimize the risk of these regulated quarantine pest introduction into new countries. Pathogens that are often symptomless in germplasm (e.g., in vitro plants, corms, and suckers), such as viruses, pose a special risk to the movement of vegetative germplasm. The Bioversity International-CIAT Alliance (1617 accessions), and the IITA (393 accessions) germplasm collections are managed as in vitro cultures. It has been shown that bacterial and fungal contaminants in banana shoot tip culture can be eradicated by isolating small explants, e.g., 1 mm meristems, and culturing them in vitro, but the virus infection still presents an important risk. To mitigate these risks, the Conservation Thematic Group of MusaNet, an international network for *Musa* genetic resources coordinated by Bioversity International, has recently edited a new version of technical guidelines to minimize the risk of pest introductions, through the movement of germplasm [58]. These guidelines followed a recommendation issued on the basis of an analysis of the phytosanitary procedures carried out by GHUs [56]. As per the new guidelines, at least four plants for each accession are grown for six months in a greenhouse. Leaf sampling is carried out from the limb and midrib of the three youngest leaves after 3 and 6 months for the comprehensive detection of the five most important viruses by PCR/RT-PCR: BBrMV, BBTV, banana streak virus (BSV), banana mild mosaic virus (BanMMV), and cucumber mosaic virus (CMV). Comprehensive indexing using electron microscopy is also conducted to search for any viral particle. Sanitation of the virus-infected banana accession is a complex process requiring a combination of meristem culturing, thermotherapy, and chemotherapy. Despite numerous efforts and the continuous optimization of the protocols, the success rate of banana sanitation is around 70%. An accession indexed negative is added to an in vitro banana collection for further safe propagation and distribution. All precautions are taken to avoid any further infection to in vitro plants that could arise if the plant is transferred to the field or greenhouse before distribution.

One of the major challenges for banana germplasm exchange was posed by the finding of an integration of the BSV genome, termed the eBSV (endogenous BSV), in the *M. balbisiana* genome, which contributes to the B genome. The eBSV can spontaneously release infectious particles, especially following in vitro culturing and interspecific crosses [19]. The presence of infectious eBSVs within B genomes has emerged as a main constraint for health indexing and safe *Musa* germplasm transfers. Plants apparently negative to BSV could spontaneously become positive with the expression of the eBSV sequence. The discovery of this phenomenon in bananas in the 1990s halted banana germplasm distribution from CGIAR centers. However, the Inter-African Phytosanitary Council, the Regional Plant Protection Organization of Africa, made a provision allowing the distribution within Africa of virus-free banana and plantain that may carry eBSV [19]. Based on this regulation, the IITA genebank and breeding programs distribute virus-free banana germplasm within Africa, with the informed consent of the recipients. However, the advancement of technology and knowledge on viruses integrated in host genomes provide a way to overcome this natural bottleneck to germplasm distribution. First, diagnostic techniques were established to distinguish eBSV and episomal virus particles for virus indexing purposes; secondly, molecular markers were established to identify *Musa* accessions with activatable eBSV; and lastly, a decision model was developed to enable the distribution of *Musa* germplasm with eBSV sequences based on the consent of the importer [58].

*Cassava:* Cassava is cultivated for tuberous roots and is traditionally propagated using stem cuttings. The crop is conserved in field collections and in vitro. The in vitro collection of 6500 accessions of cassava at the CIAT in Colombia, and 3700 accessions at the IITA in Nigeria are the largest cassava ex situ collections. The germplasm is exchanged as in vitro plants and botanic seed. Viruses and phytoplasmas pose a major threat to cassava distribution as in vitro plants. A diverse range of viruses infect cassava in Latin America, Africa, and Asia (Supplementary Table S1) [59,60]. The sanitary testing of the cassava collection held by the CIAT in Colombia checks for viruses prevalent in the region: Cassava common mosaic virus (CsCMV), cassava virus X (CsXV), and four other viruses that are associated with the cassava frogskin disease: the cassava frogskin associated virus (CsFSaV), cassava polero-like virus (CsPLV), cassava new alphaflexivirus (CsNAV), and cassava torrado-like virus (CsTLV) [60]. The sanitary testing of the in vitro African cassava collection conserved in the IITA, Nigeria, mainly checks for viruses prevalent in Africa: African cassava mosaic virus (ACMV), a complex of East African cassava mosaic viruses (EACMVs), and its strains, cassava brown streak ipomoviruses (CBSIVs), and 16Sr Phytoplasmas [57]. The cassava collection in Asia mainly focuses on viruses (Indian cassava mosaic virus (ICMV), and SLCMV), and phytoplasmas prevalent in the region. Both the CIAT and IITA GHUs have the diagnostic capability to test for all viruses known to infect cassava.

Several procedures have been established for virus and phytoplasma detection to generate virus-free planting material from meristem cultures in vitro, with or without thermotherapy, chemotherapy, or cryotherapy. The basic procedure includes a heat treatment applied to of stem cuttings with a length of about 30 at 28 and 38 °C for 6 h in the dark and 18 h in the light in an incubator [43]. Apical shoots from stem cuttings, after sanitation with 3% sodium hypochlorite, are used for meristem excision and in vitro plant development. About 2- to 4-month-old in vitro plants are virus indexed by PCR or RT-PCR to detect and eliminate virus-infected plants, and the remaining plants are re-indexed second time after 3 to 4 months to ascertain their health status. The virus-free plants are used as a mother stock for conservation as ‘clean stock’, and further propagation and use. It takes about 6 to 12 months to generate a virus-free stock of cassava germplasm. These procedures are known to be robust and 90% efficient in eliminating viruses. Only virus-free in vitro plants are transferred for propagation purposes. Cassava germplasm distributed as botanic seed poses little risk of virus spread, as none of the viruses reported to infect cassava have been detected in seedlings. Nonetheless, the cassava botanic seeds are surface sterilized with insecticides and pesticides, and they are germinated in screenhouses for physical inspection, before the seedlings are released to the end-users. Occasionally, cassava germplasm is transferred as stem cuttings generated from virus-free in vitro plants under insect-proof screenhouse conditions, after stem treatment with a slurry of insecticide and fungicide cocktail to eliminate microorganisms and arthropod pests.

*Potato:* Potato is propagated through tubers. Besides viruses, viroids, and phytoplasmas, field-produced tubers can transmit a long list of bacterial, fungal, and nematode diseases, including brown rot (*Ralstonia solanacearum phylotype IIB*), softrot (*Pectobacterium* and *Dickeya* spp.) ringrot (*Clavibacter michiganensis* subsp. *sepedonicus*), wart (*Synchytrium endobioticum*), common scab (*Streptomyces* sp.), powdery scab (*Spongospora subterranea* f. sp. *subterranea*), late blight (*Phytophthora infestans*), nematodes, and insects. Potato is known to be infected by more than 50 different viruses, but only about a handful of them (PVY, PVX, PVS, PVA, and potato leaf roll virus) are significant pathogens globally; however, some unique local viruses can be of major concern [61]. The CIP maintains one of the world’s largest in-vitro genebank collections with over 7209 accessions of potato, many of which are of a local origin. The CIP uses a ISO/IEC17025 accredited process for ensuring that in vitro plants are free of all pathogens, both known and unknown. The process includes a combination of an antibacterial treatment before and during in vitro introduction, followed by virus indexing, which combines ELISA (9 viruses), RT-PCR (1 virus and 1 viroid), and a biological indicator host infection for 11 species. This indexing is repeated twice, before and after thermo-therapy and meristem tip culturing. Due to the extremely contagious and stable nature of PSTVd all plants are tested for this viroid before even starting the process of introduction and are destroyed immediately if they are found to be positive. The protocol for virus cleaning at the CIP is 91% efficient, and the most difficult viruses to clean are PVS and PVT. Only material that has been certified to be free of any pathogens after this process is permitted to be moved internationally. Any germplasm received from other institutions or countries will be tested in a similar way, before it can be made available for further distribution. Despite the rigorous indexing process, additional diagnostic tests are performed for pathogens when demanded by the importing country. Breeders occasionally move true seed and pollen internationally, and this is generally considered to be safer than moving plants or organs around. For true seed and pollen, the procedure includes testing both parents (female and male) at the pre-flowering stage for seed-transmitted viruses (APLV, APMMV, AVB-O, AMV, PVT, and PYV) and PSTVd, for which they must be negative. The seeds and pollen must be free of any insect pests and treated at −70 °C for seven days, if contamination is suspected. The seeds are surface sterilized with 2.5% sodium hypochlorite for 10 min to kill any seed-borne pathogens.

*Sweetpotato:* Sweetpotato is traditionally multiplied through stem (vine) cuttings. Like other VPCs, viruses are the principal concern for vegetatively propagation. More than 30 viruses have been reported to infect sweetpotato, but only a handful of them are of global significance and include crinivirus, sweet potato chlorotic stunt virus (SPCSV), the potyviruses, sweet potato feathery mottle virus (SPFMV), sweet potato virus C (SPVC), -G (SPVG), and -2 (SPV2), and several related begomoviruses (Supplementary Table S2) [62,63]. The CIP genebank holds 8,054 accessions of sweetpotato, and like potato, CIP has a ISO/IEC17025 accredited process for ensuring that in vitro cultivated sweetpotato are free of all known pathogens. The process is similar to that described for potato, except that testing is conducted for sweetpotato-specific viruses by PCR (begomoviruses) and ELISA (10 viruses), and that the biological indicator host range is replaced by a single universal indicator host, *Ipomoea setosa*, on which sweetpotato accessions are grafted. The efficiency of the current virus clean-up protocols for sweetpotato is 69%, and the difficult viruses to eliminate are begomoviruses, SPFMV, and SPVG. As for potatoes, only in vitro plants that have been confirmed to be free of known pathogens are distributed internationally. Sweetpotato pollen or seed is not commonly moved internationally by the CIP or partners, but the procedure would be similar to that for potato. Only two sweetpotato viruses, a begomovirus (sweet potato leaf curl virus), and a mastrevirus (sweet potato symptomless virus 1), have been reported to be seed-transmitted.

*Yam*: Unlike other clonal crops, multiple species of yam (*Dioscorea* spp.), which originated from different parts of the world, have been domesticated for the production of underground edible tubers, and the crop is traditionally propagated using tubers [64]. Out of about ten popularly grown species, *D. rotundata* (white yam) of West African origin, and *D. alata* (water yam) of Asiatic origin are widely cultivated in the world. The IITA genebank in Nigeria holds the world’s largest in vitro yam collection [64]. The global yam germplasm collection comprises about 5839 accessions of about 10 species, including *D. rotundata*, *D. alata, D. bulbifera, D. cayennensis, D. dumetorum, D. esculenta,* and a few other species. Yam is known to be infected by over 15 viruses [57,65], including yam mosaic virus (YMV), CMV, yam mild mosaic virus (YMV), several badnaviruses, generically referred to as yam bacilliform viruses (YBVs), Japanese yam mosaic virus, Chinese yam necrotic mosaic virus, yam asymptomatic virus 1, yam virus Y, yam chlorotic necrosis virus (YCNV), yam chlorotic mosaic virus (YCMV), Dioscorea mosaic-associated virus, and air potato ampelovirus 1. Of these, YMV is known to cause the most economic damage in *D. rotundata* and *D. cayanensis* worldwide, whereas YCMV and YCN are important for Chinese and Japanese yam in Asia. The other viruses detected in yam either cause mild mottling or no symptoms at all. Many yam viruses are not regulated, although the IITA uses protocols to generate virus-free in vitro plants for conservation and distribution [66]. Virus elimination is achieved by selecting asymptomatic plants for thermotherapy and regeneration of in vitro plants from meristem explants. The in vitro plants are subjected to virus indexing using PCR-based methods, after three months of post-flask growth, to ascertain their health status. Plants that test negative are bulk propagated for conservation and distribution. The integrated viral sequences of badnaviruses have been detected in yam genome sequences, but unlike in eBSV in bananas, the endogenous badnavirus sequences in yam are defective and not known to generate infectious particles. They were therefore not regarded to have any phytosanitary significance. Compared to other VPCs conserved by the CGIAR, yam phytosanitation is a lengthy process and usually takes between 12 to 24 months. Of the various associated factors, the slow in vitro meristem growth is a major bottleneck for the production of virus-free plants. True seed of yam is exchanged by breeding programs after seed treatment with a fungicide and an insecticide. Tubers generated from virus-free yam plants are also used for international exchange, especially within the West African sub-region, by breeding and seed system initiatives.

### 4.3. Trees

*Germplasm conservation approach*. The World Agroforestry Centre (ICRAF) characterizes and conserves 20,000 accessions of over 200 species of agroforestry trees, both indigenous and exotic species for agroforestry and restoration programs in Africa, Asia, and Latin America. Germplasm is conserved as seeds in genebanks or adult trees in field genebanks, where provenances are grown and evaluated in common gardens for domestication and cultivar testing [67]. Due to the wide range of plant growing conditions and geographic locations, this conservation approach implies that tree germplasm is exposed to a plethora of seed-borne pathogenic organisms, including both native and non-native microorganisms and insects. Furthermore, the vast taxonomic and geographic diversity of collected germplasm is susceptible to being impacted by poorly characterized, or unknown pests and diseases [68]. This scenario poses a major challenge in the implementation of phytosanitary measures for the detection and mitigation of pests and diseases of tree germplasm. Plant health interventions were put in place through collaborative research with the NPPO. High priority tree taxa were selected based on the strategic importance of the crop (food, timber, and non-timber product, and biodiversity conservation), and major biotic threats were determined based on laboratory testing of seed and vegetative tissue and field monitoring. Prioritized species include relevant food and multipurpose trees native to Africa, such as baobab (*Adansonia digitata*) and marula (*Sclerocarya birrea*), or exotic trees, such as Southern silky oak (*Grevillea robusta*) and eucalypts (*Eucalyptus* spp.). Special efforts have been made to characterize the taxonomy and pathogenicity of emerging fungal diseases of an unknown origin within the Botryosphaeriaceae family, which have the potential of impacting multiple tree species in Africa [69]. Standard operating procedures (SOPs) for assessing the germplasm health of both seeds and trees in the open field were developed and include: (a) seed health testing for the detection of seed-borne pathogens; (b) field assessment of incidence and damage caused by tree cankers, (c) detection and identification of tree canker fungi; and (d) field assessment of incidences and damage caused by fruit and seed insect pests. These procedures are to be used routinely as phytosanitary measures to evaluate germplasm prior to exchange or collection and for monitoring plant health in the field.

### 4.4. Forages

The CIAT and ILRI genebanks hold 22,694, and 18,662 accessions of forages (cereals and legumes), which include over 1400 species, the vast majority of which are wild species [5]. Most forage accessions are distributed as seeds, except for a few that rarely produce seeds, such as Napier (or elephant grass, *Pennisetum purpureum*), which are maintained by vegetatively propagation in field genebanks. The regeneration of forage germplasm faces a great variety of growth habits and development times, due to the large number of genera, as well as the considerable number of species, belonging to the same genus. All of this makes it difficult to use standardized health testing measures on a large scale. For this reason, most of these materials are regenerated in the open field and occasionally under a screenhouse, making use of the integrated pest and disease control and management of diseases expensive. Except for a few widely used species, the knowledge on pests and diseases is limited for rigorous health testing. Most countries lack sufficient records of the pests and diseases affecting forages, which complicates international germplasm transfers. As in the case of tree germplasm, strict integrated phytosanitary control measures are used to maintain germplasm health, but much work is still needed to generate baseline knowledge on seed-borne and seed-transmitted risks in order to develop robust procedures for safe exchange.

## 5. GHU Support for CGIAR Programs

### 5.1. Enabling Safe Germplasm Transfers

**Figure 3:**
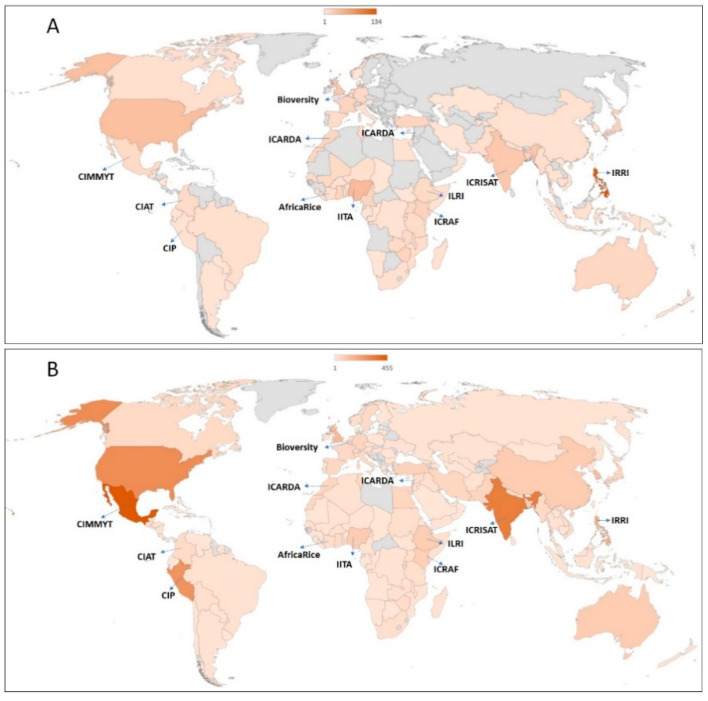
Countries from which the CGIAR centers received germplasm (**A**), and countries that received germplasm from the CGIAR centers (**B**) in 2018–19. The data combine transfers from the genebanks and crop breeding programs. The intensity of the orange shade indicates the number of transfer instances.

The GHU operations are demand-driven and accommodate the evolving needs of the genebanks and breeding programs. The centers export and receive thousands of germplasm samples from genebanks, breeding programs, and elite seeds of released cultivars for evaluation and use by national and international partners [5,6,7]. Over 80% of the CGIAR centers’ germplasm exports are executed mainly from twelve countries, Belgium, Colombia, Cote d’Ivoire, Ethiopia, India, Kenya, Lebanon, Mexico, Morocco, Nigeria, Peru, and Philippines. All of these countries host the headquarters, main genebanks, and breeding programs of the centers, except for Belgium and Morocco. The remaining CGIAR centers’ international transfers and/or regeneration activities are operated from about 12 to 16 countries, including Benin, Cameroon, Mali, Malawi, Tanzania, Turkey, Uganda, Vietnam, Zambia, and Zimbabwe, which also host the CGIAR breeding programs, and ex situ and in situ collections of genebanks. Germplasm exports from the CGIAR centers located in these countries cater for between 90 and 130 countries per year, in all continents (Figure 3).

**Figure 4:**
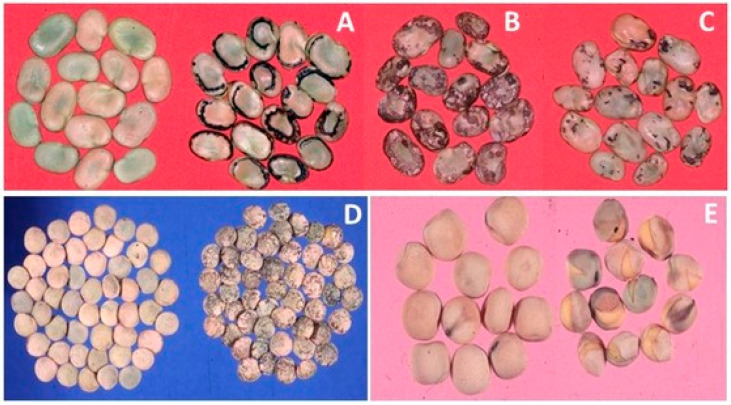
Symptoms of necrosis, size reduction, and malformation in seeds of faba bean, virus-infected (right) and healthy (left): causal viruses are broad bean stain virus (BBSV) (**A**), broad bean mottle virus (**B**), and bean yellow mosaic virus (**C**). Symptoms of necrosis in lentil seeds caused by BBSV (**D**), virus-infected (right), healthy (left). Cracking caused by pea seed-borne mosaic virus (PSbMV) in pea seed (**E**), virus-infected (right), healthy (left).

**Figure 5:**
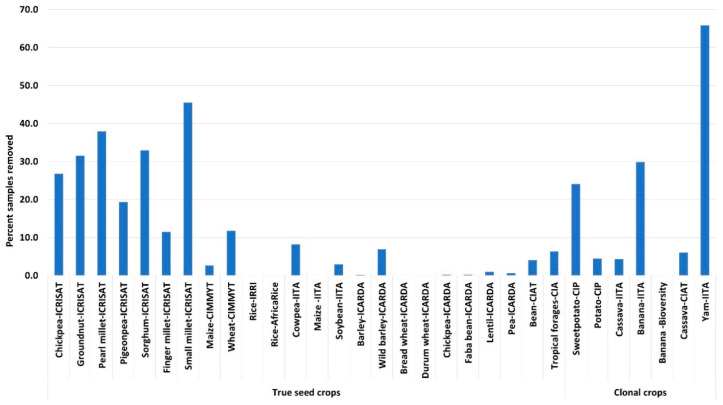
Percentages of samples removed, due to pest interception during phytosanitary processing.

Uniform standards are applied to all export and import events for successful pest-free germplasm transfers. For instance, in 2018 and 2019, GHUs facilitated 1300 and 2600 events of germplasm transfers from genebanks and breeding programs, respectively, to 150 countries (Figure 3) (Supplementary Figure S1). This onerous task involves the production and extensive testing to ascertain the health status of germplasm released to end-users. In 2018 and 2019, GHUs tested and removed 7% of the 335,928 genebank samples, including those for import, export, and regeneration, and 3% of the 118,044 breeding samples for import and export, were found to be infected with pests (data not shown) [5]. In this process, a total of 2.47 million diagnostic reactions were employed to analyze the 453,972 samples in the two years at an average annual cost of about US$ 12 million. The proportion of infected samples detected varies by crop, and the pests most frequently detected are endemic in regeneration sites. An example set of some pest-infested legume seeds are shown in Figure 4, and the percentages of rejections during the phytosanitary testing of crop germplasm in 2019 are shown in Figure 5. The infected samples are returned to the phytosanitary treatment cycle or replaced with healthy stock, and the infected samples are then subjected to incineration. The data and knowledge from extensive phytosanitary surveillance of germplasm helped GHUs to make improvements to their procedures and protocols, some of which have played a vital role in confirming the first occurrence of regulated pests in new territories [15,16,17]. The technical resources and skill set of GHUs also support the centers’ and partner initiatives in combating emerging pests, suppling of reference material for diagnosis and phenotyping, developing national program capacity, and improving awareness and advocacy associated with transboundary pest prevention and control.

### 5.2. Partnerships Enabling GHU Functions

Excellent partnerships and special arrangements, both formal and informal, between the GHUs and NPPO of the host countries, and regional plant protection organizations (e.g., Inter-African Phytosanitary Council in Africa; and Comunidad Andina de Naciones (CAN) in South America) have a significant role in enabling the successful exchange of germplasm. Such arrangements enabled the establishment of special facilities for CGIAR germplasm processing. For instance, the National Bureau of Plant Genetic Resources (NBPGR), which is responsible for the quarantine monitoring of germplasm exchanges from India, established its Regional Station at Rajendranagar, Hyderabad, India, in 1986 as the sole plant quarantine authority for clearing the germplasm and breeding material of ICRISAT’s mandate crops [54]. Similarly, special arrangements exist between the CIAT and the Colombian Institute of Agriculture (ICA) in Colombia; CIP and Servicio Nacional de Sanidad Agraria (SENASA) in Peru; the CIMMYT and El Servicio Nacional de Sanidad, Inocuidad y Calidad Agroalimentaria (SENASICA) in Mexico; the IRRI and the Bureau of Plant Industry in Philippines; the ICARDA and the Plant Protection and Plant Quarantine Department of Lebanon; the IITA and the Nigerian Agricultural Quarantine Services (NAQS) in Nigeria; the ICRAF, IITA, CIP, and CIMMYT and Kenya Plant Health Inspectorate (KEPHIS) in Kenya, to name a few. Close partnerships also exist between non-NPPO agencies, for example, the relationship between the Alliance of Bioversity International-CIAT and the University of Liege (Belgium), and the ICRAF and the Kenya Forestry Research Institute (Kenya), which enable the centers’ GHU activities. These arrangements between various partners recognize GHUs as part of the national diagnostic facilities, undertake collaborative research on phytosanitary issues and pest surveillance, jointly organize national and regional phytosanitary capacity development events on emerging disease control, and undertake awareness and advocacy activities to improve phytosanitary practices, policies, and regulations.

### 5.3. GHUs in Capacity Development

The GHUs play very important roles in the training of personnel, institutional capacity building, and raising awareness at a global level. The GHUs organize at least 10 workshops each year for staff from national and regional organizations on various phytosanitary themes, including diagnostics, seed health testing, and seed treatment. Since 2017, GHUs have organized “The International Phytosanitary Awareness Week” in coordination with the NPPOs, RPPOs, and IPPC [70]. These activities have the objective of informing interested parties about GHUs and their role in the secure distribution of germplasm, as well as the importance of pathogens and pests in crop production. The week-long event also aims to secure links between institutions through the consolidation of a “Community of Practice”, fostering collaboration and exchange in such areas as joint projects, technical-scientific support, and capacity building, among other areas, with the goal of providing a ‘front line’ free-flow of information to assist in decision-making and facing upcoming challenges. These meetings bring together representatives of institutions from the public sector and academia, as well as staff, to take part in a variety of activities based on a main theme, which is different every year. For instance, the 2020 theme focused on *“phytosanitary safety for transboundary pest prevention”* to mark the United Nations International Year of Plant Health 2020 (IYPH 2020) [71]. These activities increase awareness of the importance of phytosanitation locally and globally and create a scientific forum for raising awareness of the phytosanitary challenges and organizational responsibilities associated with ensuring the distribution of healthy seed and sustainable agricultural production that will contribute to the global fight against hunger and malnutrition.

## 6. Challenges and Opportunities

### 6.1. Evaluation and Reevaluation of Germplasm Health

The comprehensive phytosanitary testing procedure used to declare the health status of an accession and its suitability for safe distribution or conservation is a tedious and time-consuming task (about 6 to 24 months for clonally propagated crops and 3 to 6 months for true seed crops). Untested accessions or accessions that fail health tests are marked as “unavailable for distribution”. For instance, about 75% of the true seed accessions of staple crops held in ex situ collections of the CGIAR genebanks have been health certified and are available for distribution, compared to about 55% of clonally propagated accessions [5]. However, the discovery of new pests in a crop species sometimes necessitates a reevaluation for the detection of the newly reported pests and recertification of the health status of accessions and their availability for distribution. For instance, the discovery of new viruses and a phytoplasma with the cassava frogskin disease etiology in Colombia [60], led to a reevaluation and health certification of the in vitro cassava collection held at the CIAT in Colombia. Similarly, the discovery of causal viruses of cassava brown streak disease (CBSD) in 2002, and the CBSD outbreak in the Greatlakes region of Eastern Africa in 2008 [57] led to a precautionary evaluation of the in vitro and field collection of cassava conserved at the IITA Ibadan station in Nigeria. The outbreak of MLN in Eastern Africa in 2011 affected the distribution of maize germplasm in Eastern Africa, which was resumed after the establishment of procedures for the reliable detection of maize chlorotic mottle virus (MCMV) in the seed lots and phytosanitary procedures for the safe production of maize seed [42]. While instances of this type are infrequent, they result in a significant impact on germplasm distribution and result in additional costs. At the same time, these instances also aided GHUs in gaining experience in quickly adapting to new pest situations and establishing optimal protocols for phytosanitation and diagnostics to restore phytosanitary protection of germplasm and field crops. It also helped GHUs to engage with national, regional, and continental efforts to control epidemics caused by introduced transboundary pests or the emergence of new strains or species of an established threat.

### 6.2. Variable Standards and Different Phytosanitary Demands

GHUs work on different crops, wild relatives, and pests in various countries. Therefore, the variety of needs arises from the different phytosanitary statuses of the crops in each geography, the technologies available for detection, diagnosis, and phytosanitation, and the standards adopted by the NPPO in the country of operation [45]. For instance, the import conditions for cassava between Nigeria and Ghana are different from that between Nigeria and Vietnam, due to different pest risks. The standards for germplasm distribution from genebanks are not always well established. The NPPO adopts ISPMs designed for commercial consignments of plants and plant products, with specific modifications of their own for dealing with small sample sizes distributed from genebanks. Due to the better knowledge of pest risks, the standards for some crops are relatively well defined (e.g., banana, bean, cassava, cowpea, chickpea, groundnut, maize, potato, rice, wheat, and other crops) [47]. However, the vast taxonomic range, geographic diversity, and limited knowledge of the pest risks to crop wild relatives, trees, and forages pose significant challenges in the implementation of appropriate testing standards for pest detection. To overcome some of these challenges, GHUs began developing harmonized Quality Management Systems (QMS), termed the GHU-QMS, to achieve uniform standards across GHUs. The GHUs of the CIAT, CIMMYT, and CIP are ISO/IEC17025 accredited for the quality assurance of seed health testing methods. As of 2019, 139 SOPs have been developed, with 7 to 30 per GHU, depending on the center and country. These procedures were introduced with the aim of having GHUs conform uniform standards by the end of 2021.

The implementation of phytosanitary measures and policies for tree germplasm critically lacks in Africa. The extraordinary taxonomic and geographic diversity of the tree germplasm collected, and the availability of field genebanks, in addition to seed banks, show an opportunity for boosting the detection and characterization of emerging pathogens in line with the “sentinel plant” [72]. This should fuel fruitful collaborations with the NPPOs and IPPC and contribute to the much-needed updating of the lists of quarantine pest and diseases of tree species.

### 6.3. Changes in Pest Dynamics

The changes occurring in the dynamics of pests have a significant impact on germplasm transfers from the centers. Several economically important pest outbreaks in the last decade were attributed to introduced pests, as explained in the previous sections [14]. The perception of pest risk is also influenced by the severe destruction caused by unrelated pest outbreaks. For instance, the olive decline caused by introduced *Xylella fastidiosa* in Italy, citrus greening caused by *Candidatus liberibacter* spp., in the USA, and several other examples, including the Covid-19 pandemic, have a significant influence on the regulatory procedures and decision-making relating to germplasm transfers [13]. In addition, the discovery of new virus species using novel diagnostics technologies is adding to the burden of risks from the pests that are already known [73,74]. A study estimated that many alien pests introduced into countries are yet to be detected [13], a status termed “pseudo-absence”, which implies the potential occurrence of a pest in the geography, but apparently considered there to be no alien pests because none had been found. This familiar but unquantified risk of “known-unknowns” and “unknown knowns” is a major threat for international germplasm exchange programs, which relies on pest occurrence knowledge in the country of the germplasm origin.

Over the years, GHUs have adjusted to changing pest dynamics, including undetermined pest risks and have taken adaptive measures to sustain operations. Following the MLN outbreak in East Africa, the CIMMYT GHU team established sampling and treatment procedures to sustain maize germplasm transfers. Similarly, the IITA-CIAT established cassava virus elimination protocols to maintain germplasm transfers between continents, including the use of transit centers for intermediary evaluation before delivering material to a final destination. Recently, the ICRAF GHU documented the invasive pests of native African tree germplasm, conserved as a resource for updating pest lists [69]. Procedures for the health testing of true seed crops from seedling to harvest, and a seed health test offers robust measures for the detection of both known and new pathogens. Clonal crops are more complicated, especially cryptic and latent viruses, which do not induce any symptoms and avoid detection. To overcome these challenges, GHUs have adopted HTS technologies and the bioinformatic reconstruction of viral sequences, which make it conceptually feasible to detect any viral agent by HTS of the nucleic acids from a host and the identification of viral sequences of known or unknown agents in the generated sequences [73,74]. These developments will strongly impact the way virus diagnostics is performed in the coming years. A pilot project focusing on the application of HTS technologies to improv the virus indexing of clonal crop germplasm accessions has been initiated for bananas, cassavas, potatoes, sweetpotato, and yams at the Bioversity International, the CIAT, the CIP, and the IITA.

### 6.4. Keeping up with Evolving Technologies

New technologies are evolving all the time for the phytosanitation and more accurate and rapid detection of existing and newly diagnosed pests. GHUs maintain a balance in adopting the best technologies that offer cost and time efficiency, meet regulatory requirements, and comply with ISO/QMS systems. The GHU operating system supports the use of a well-standardized procedure, so long as the procedure remains effective and offers reliable results for decision-making. The development of new standardized procedures is expensive, time-consuming, and requires extensive testing under various scenarios to determine the robustness, reliability, and suitability of the new method for the intended purpose. GHUs aim at keeping up to date and staying relevant, while avoiding change for the sake of change. As an example, the GHUs use, HTS-based diagnostic methods in the phytosanitary context is limited to virus indexing of mother stocks, while PCR and ELISA-based methods remain as ‘gold standard’ for virus indexing.

GHUs have identified a need to intensify efforts towards developing nucleic acid-based detection protocols for several pests that are difficult to detect through routinely used conventional tests, such as the blotter technique. Efforts are also required to standardize protocols for non-invasive techniques for detecting seed-borne pests (e.g., Videometer spectral imaging for detecting fungal pathogens and soft X-ray analysis to detect hidden seed infestation by pests) [75]. Similarly, new and safe solutions for crop protection and seed treatments are needed, as some fungicides and insecticide treatments are banned or restricted for use on specific crops in some countries. Due to the high volume of samples processed annually, the adoption of mobile digital data collection devices is necessary to facilitate the processing of materials, which would notably improve the traceability of the process and real-time data collection and analyses.

### 6.5. Insufficient Phytosanitary Standards for Germplasm Transfers from Genebanks and Breeding Programs

Specific phytosanitary standards for the international exchange of germplasm have not been developed. The FAO Genebanks Standards [35] lack adequate details on the procedures for the import and export germplasm from international genebanks. Therefore, the NPPOs either develop and follow their norms or follow those prescribed through ISPMs, which were established to address the SPS regulations governing the trade of plant and plant products, as part of the WTO treaty [34]. To date, the 43 ISPMs have been developed by the IPPC are aligned with the SPS requirements concerning commercial trade and large volumes of consignments. These regulations are inadequate for the purposes of the international transfer of germplasm. The ISPM 36 on the “international movement of plants for propagation” [76], and the ISPM 38 on the “international movement of seed” [77] address few issues, but are mainly designed for commercial shipment volumes. The ad hoc norms for germplasm exchange from genebanks and breeding programs introduce different requirements, depending on the country, making germplasm transfers a challenging endeavor. In addition, the existence of conflicting regulatory frameworks in different countries due to outdated regulations, outdated pest lists, or their absence constrain the exchange of germplasm. All of this causes delays in clearance, leading to germplasm having a loss of viability, before it arrives at its destinations, or a late arrival, resulting in the loss of an entire planting season.

Other challenges emerge from unforeseen changes to policies in the countries of operations. Policy changes are most often triggered by (i) new pest outbreaks, (ii) the risk perception of invasive pests spreading into territories, (iii) the introduction of new/amended procedures, and (iv) changes to administrative and implementation protocols. GHUs have adopted the flexibility needed to make necessary adjustments in order to align with policy requirements in countries of operations and thus enable germplasm distributions. In some cases, the policies do not match the biological complexities and restrict germplasm distributions. For instance, the genomes of some viruses are integrated into the host genomes (e.g., endogenous badnavirus sequences in banana and yam genomes). In essence, integrated viral genomes are an inseparable part of the host. The existing regulations do not consider these complexities, and all the germplasm with integrated virus genomes was consequently withheld from international transfers, and this amounts to over 50% of the *Musa* collection held in the CGIAR genebanks. In 2015, GHUs of IITA-Bioversity, together with the MusaNet Working Group on Genetic Resources, developed a new approach to the transfer of germplasm with integrated viral sequences (see Section 4 for details) [58]. The NPPOs have approved international transfers of *Musa* germplasm organized in accordance with this protocol, making a significant proportion of banana germplasm available again for distribution.

The current phytosanitary policies are also insufficient to address the germplasm “safety duplication” efforts (also referred to as black box conservation) in the Svalbard Global Seed Vault and/or in other third-party countries [5]. The safety duplication involves transfers of both health certified and untested accessions to another country (third-party) in sealed envelopes or as in vitro plants exclusively for conservation and repatriation to the “country of origin” when required. The NPPO requirements, however, are difficult to fulfill, as the procedures stipulate mandatory health declarations, and the entry of untested germplasm is prohibited. However, ad hoc bilateral arrangements have been established between the source of origin countries and third-party countries to facilitate safety duplication as an interim arrangement. This system is working although not always smoothly due to ambiguities arising from the different understandings of the NPPOs. GHUs are working with regulatory agencies to establish a standard policy to streamline the procedure for this important genebank activity.

To cover some of the phytosanitary policy challenges associated with germplasm exchange, GHUs have initiated the development of the “CGIAR GreenPass Phytosanitary Protocol (GreenPass)” [78], as a comprehensive procedure for the assurance of phytosanitary compliance. This protocol will detail the best procedures in use for germplasm regeneration and health assurance, while maintaining transparency in risk assessment and mitigation strategies to obtain NPPO accreditation in order to fast track germplasm distribution. It is hoped that the IPPC and other stakeholders’ endorsement of this initiative will eliminate redundant checks or reduce the processing time of material from GreenPass-accredited facilities.

## 7. Conclusions

The CGIAR germplasm health program has over 50 years of experience [41]. GHUs have served as a vital conduit of the globally coordinated CGIAR crop research programs, which tested 1000s of germplasms and new breeding lines in multiple field sites and mega environments for the identification of lines that have superior yields, high nutrition and are resilient to biotic and abiotic stresses. The seeds of those accessions were made widely available for crop productivity improvement, leading to a broad social, economic, and environmental impact [79,80,81]. For instance, the International Wheat Improvement Network (IWIN) organized approximately 700 field sites in over 90 countries to develop around 1000 high-yielding, disease-resistant lines targeted at major agro-ecologies, which are delivered annually as international public goods (IPGs) [81]. To date, GHUs continue to facilitate crucial germplasm transfers to the largest number of stakeholders around the world vital to deliver IPGs with a positive impact on the SDGs associated with (i) nutrition and food security; (ii) poverty reduction; (iii) environmental health and biodiversity; and (iv) climate adaptation and greenhouse gas reduction.

The efforts of GHUs in thoroughly testing germplasm accessions for known pests, before their release for international transfer, have averted the inadvertent spread of quarantine pests. This is of great significance, as most CGIAR centers operate in countries where some of the most dreaded pests are prevalent (e.g., cassava brown streak virus, Karnal bunt, maize lethal necrosis, rice blight, and wheat blast, to name a few) [82]. Years of experience indicate that adaptability is a vital requirement for sustaining operations in an era of constant changes driven by pest outbreaks, agricultural intensification, climate variability, phytosanitary policies, and regulations [83]. A study on the patterns of invasion and spread pathways of 1517 invasive species reported that horticulture and the nursery trade are the dominant pathways for the incursion of invasive alien species [84]. The increasing international exchanges and the globalization of the world present a high risk that introduced pests will be established and expand quickly [82]. Safe and efficient germplasm transfer forms a critical preventive pest control approach for the CGIAR programs under the IPPC treaty and national laws. It is also safe to assume that the drivers responsible for transboundary pest outbreaks are difficult to contain, and high levels of vigilance will be required to monitor the pest dynamics in order to sustain the CGIAR operations. This requires regular updating of the existing protocols for hitherto unknown pests, enhanced collaboration with phytosanitary organizations and academia to obtain the most advanced information on pest detection and epidemiology, and adequate funding support, which is necessary for continuous adaptation to new pest challenges. It is imperative for GHUs to leverage technological advances in diagnostics, ICTs, remote sensing, and modeling to predict and monitor pest dynamics at a global level in order to understand their dispersal mechanisms and impact on the genebank and breeding programs in the short, medium, and long term.

GHUs high-level capacity, experience, track record, and global distribution in the developing world enable them to play an important role as centers of excellence in supporting national and regional pest and disease surveillance and rapid response. A strong case exists for positioning GHUs as part of a global network of phytosanitary hubs for the research, diagnoses, and control of established and emerging pests as part of the One CGIAR program, which is set to be operational in 2022 [85].

## Figures and Tables

**Table 1:** Some of the most economically important disease outbreaks, caused by pests introduced into sub-Saharan Africa (SSA).

Disease	Pest	Hosts	First Detection and Spread in SSA	Spread Pathway
Asian soybean rust	*Phakopsora pachyrhizi* (fungus)	Soybean, >150 legume species	First detected in Zambia in the 1980s; spread to most of sub-Saharan Africa	Possibly spread through air-borne urediniospores, which blew from Western India to East Africa
Banana bunchy top	*Banana bunchy top virus* (virus)	Banana/plantain	First reported in Egypt in 1902; and then in the Democratic Republic of Congo; spread to most of Central Africa, and adjoining countries in Southern and Western sub-Saharan Africa	Introduced from South-Pacific or Asia through infected suckers; further spread through exchange of infected planting material and aphid (*Pentalonia nigronervosa*) vectors
Banana bacterial wilt	*Xanthomonas campestris* pv. *Musacearum* (bacterium)	Banana/plantain	Burundi, Rwanda, Democratic Republic of Congo, Uganda, Kenya, Tanzania	Possibly introduced from Ethiopia to Uganda and then across the Great Lakes region through planting material, infected plant parts, or contaminated tools
Cassava green mite	*Monoychellus tanajoa* (Insect)	Cassava	First detected in Uganda in 1971; presently widespread in Africa	Introduced from South America into Africa; path of spread not known
Cassava mealybug	*Phenacoccus manihoti* (Insect)	Cassava	First detected in the Democratic Republic of Congo in 1973; presently widespread in Africa	Possibly introduced from South America to Africa through plant parts, or containers
Cassava brown streak	*Cassava brown streak ipomoviruses* (Virus)	Cassava	Native species in Malawi, Mozambique, and Tanzania in Southern Africa; first recorded in East Africa in 2004 in an epidemic around the Great Lakes region Burundi, Comoros, DRC, Kenya, Rwanda, Uganda, and Zambia	Spread through exchange of infected stem cuttings and whitefly (*Bemisia tabaci*) vectors
Fall armyworm	*Spodoptera frugiperda* (Insect)	Maize and other crops	First detected in Nigeria in 2016; known to occur in all African countries	Path of spread not known; likely through plants, plant parts, or cargo containers.
Fruit fly	*Bactrocera dorsalis * (Insect)	Mango and other crops	First reported in Mauritius in 1996; and on the mainland in Kenya in 2003; presently widespread in Africa and offshore islands	Pathway of spread not known; likely through plants, plant parts, or cargo containers
Maize lethal necrosis	*Maize chlorotic mottle virus*	Maize, sorghum, pearl millet	First detected in Kenya in 2011; then in Burundi, DRC, Ethiopia, Rwanda, Tanzania, and Uganda	Possibly spread from Southeast Asia
Panama disease—tropical race 4	*Fusarium oxysporum* f. sp. *Cubense* Tropical Race 4 (Fungus)	Banana/plantain	First detected in 2010 in Mozambique; no reports of further spread in Africa	Introduced from South East Asia; possibly introduction through contaminated soil or planting material
Papaya mealybug	*Paracoccus marginatus* (Insect)	Papaya and several hosts	First detected in Ghana in 2009; spread to most African countries	Possibly spread from Southeast Asia /South Pacific islands through plants, plant parts, or other sources
Potato cyst nematode	*Globodera pallida* (Nematode)	Potato	First detected in Algeria in 2011; subsequently in Kenya in 2018; and then in neighboring countries in East Africa	Possibly spread from European region with seed potato
Taro blight	*Phytophthora colocasiae* (Oomycete)	Taro	First reported in Nigeria in 2011; Spread to most countries in sub-Saharan Africa	Possibly spread from South Pacific islands through infected corms
Tomato leaf miner	*Tuta absoluta* (Insect)	Tomato and other solanaceous crops	First reported from Benin in 2005; spread to most countries in Africa	Wind-borne spread of insects from the northern region
Wheat blast	*Magnaporthe oryzae* pathotype *Triticum* (Fungus)	Wheat	First reported in Zambia in 2020	Possibly spread through contaminated seed/grain

Source: CABI Invasive Species Database: https://www.cabi.org/isc/ (accessed on 29 November 2020).

## Data Availability

Not applicable.

## Supplementary Materials

The following are available online at https://www.mdpi.com/2223-7747/10/2/328/s1, Table S1: Details of crops and country locations of the CGIAR GHUs; Table S2: List of crops and pests routinely assessed by the CGIAR GHUs. Figure S1: Countries receiving germplasm from genebanks (A) and breeding programs (B); and the countries from which germplasm was received by the CGIAR genebanks (C) and CGIAR breeding programs (D), during 2018–19. The intensity of blue reflects the number of transfer events.
