# Supplementary figures and images for: Phytosanitary Interventions for Safe Global Germplasm Exchange and the Prevention of Transboundary Pest Spread: The Role of CGIAR Germplasm Health Units
Source: Plants (Basel). 2021 Feb 9;10(2):328. doi: 10.3390/plants10020328 (PMC7915052; doi:10.3390/plants10020328)

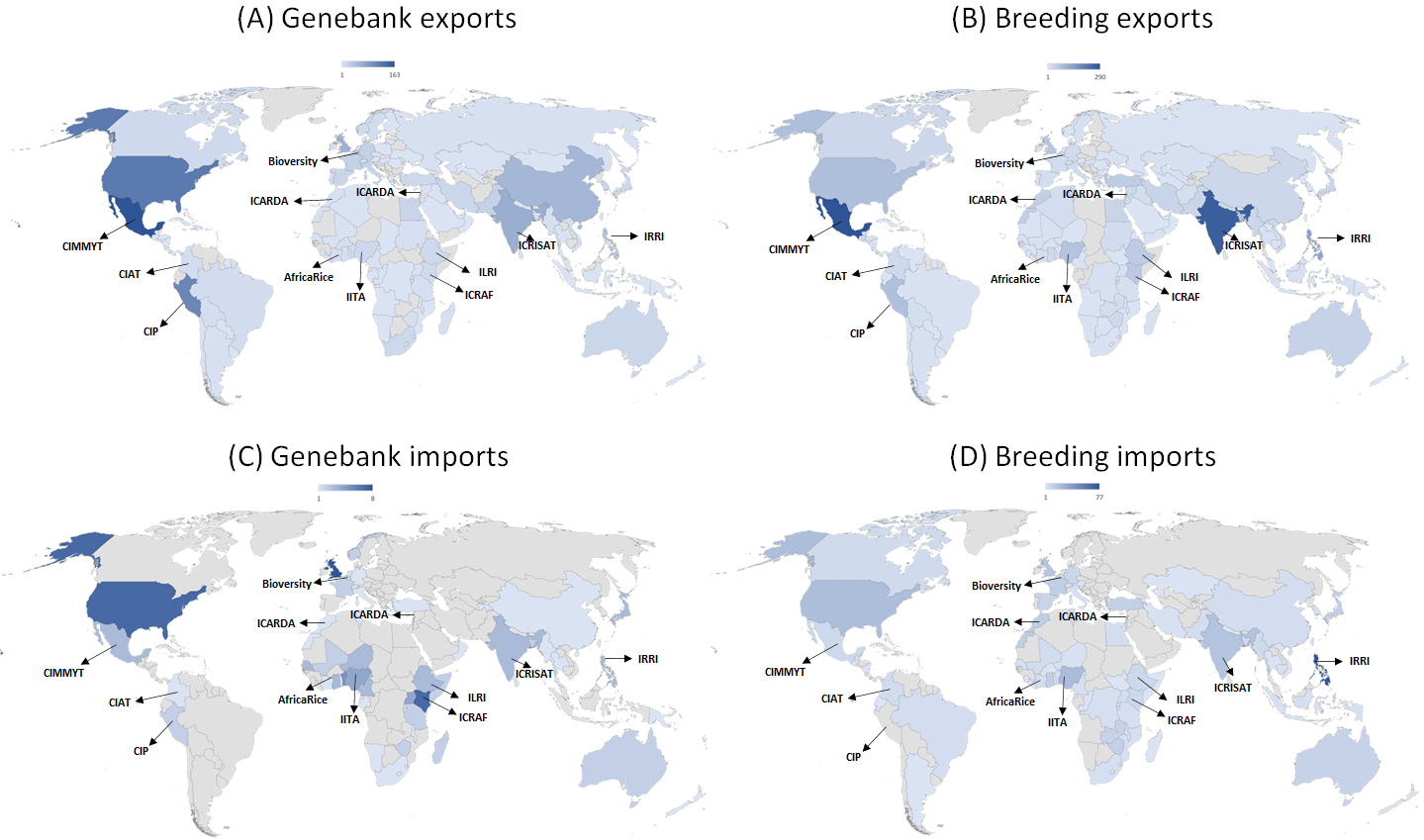

Supplement: Supplementary file 1 [file plants-10-00328-s001.zip › supple/Supplementary Fig 1 CGIAR distribution by breeding and genebanks.jpg]
